# Silver Nanowire Transparent Conductive Electrodes for High-Efficiency III-Nitride Light-Emitting Diodes

**DOI:** 10.1038/srep13483

**Published:** 2015-09-03

**Authors:** Munsik Oh, Won-Yong Jin, Hyeon Jun Jeong, Mun Seok Jeong, Jae-Wook Kang, Hyunsoo Kim

**Affiliations:** 1 School of Semiconductor and Chemical Engineering, Semiconductor Physics Research Center, Chonbuk National University, Jeonju 561-756, Republic of Korea; 2 School of Flexible and Printable Electronics, Polymer Materials Fusion Research Center, Chonbuk National University, Jeonju 561-756, Republic of Korea; 3 Department of Energy Science, Sungkyunkwan University, Suwon 440-746, Republic of Korea

## Abstract

Silver nanowires (AgNWs) have been successfully demonstrated to function as next-generation transparent conductive electrodes (TCEs) in organic semiconductor devices owing to their figures of merit, including high optical transmittance, low sheet resistance, flexibility, and low-cost processing. In this article, high-quality, solution-processed AgNWs with an excellent optical transmittance of 96.5% at 450 nm and a low sheet resistance of 11.7 Ω/sq were demonstrated as TCEs in inorganic III-nitride LEDs. The transmission line model applied to the AgNW contact to *p*-GaN showed that near ohmic contact with a specific contact resistance of ~10^−3^ Ωcm^2^ was obtained. The contact resistance had a strong bias-voltage (or current-density) dependence: namely, field-enhanced ohmic contact. LEDs fabricated with AgNW electrodes exhibited a 56% reduction in series resistance, 56.5% brighter output power, a 67.5% reduction in efficiency droop, and a approximately 30% longer current spreading length compared to LEDs fabricated with reference TCEs. In addition to the cost reduction, the observed improvements in device performance suggest that the AgNWs are promising for application as next-generation TCEs, to realise brighter, larger-area, cost-competitive inorganic III-nitride light emitters.

Transparent conductive electrodes (TCEs), which are used as *p*-type spreading contacts in III-nitride light-emitting diodes (LEDs), are of particular importance because of their direct influence on light extraction efficiency, forward voltages, device reliability, and production cost^1^,^2^,^3^,^4^,^5^,^6^,^7^,^8^,^9^,^10^,^11^,^12^,^13^,^14^,^15^. In the III-nitride LED field, one technological breakthrough in the past decade was adopting indium-tin-oxide (ITO) electrodes, because these electrodes yield a high optical transmittance (*T*_op_) (approximately 93% at 450 nm), a low sheet resistance (*R*_sh_) (~15 Ω/sq (commercial-grade)), and a feasible ohmic contact to *p*-GaN^16^,^17^,^18^,^19^,^20^. Despite ITO’s good electrical and optical properties, it is important to identify an alternative to this material for the following reasons. First, the use of ITO materials is not cost-competitive owing to the rapid depletion of the elemental indium source and the complex vacuum systems required for the deposition of ITO films. Second, ITO deposition by conventional sputtering processes results in ion damage to the *p*-GaN surface^21^,^22^, degrading the ohmic contact and causing a leakage current to evolve. To overcome this damage-related issue, conventionally, an *e*-beam evaporator and/or a modified sputtering system are used. In addition, elaborately designed *p*-contact layers, such as superlattices^23^,^24^ and tunnel junctions^25^,^26^, have been employed because of their reduced sensitivity to the ion damage arising from the use of an *n*-type conductive top layer. However, these methods suffer from the relatively poor electrical and optical properties of deposited ITO films, the complex vacuum systems, and the increased epitaxial growth sequences and efforts. Third, although the *T*_op_ of ITO films in the visible wavelength is high enough to be usable, it is quite poor at the deep ultraviolet (UV) wavelength of ~260 nm (owing to the band-to-band absorption by ITO), which is regarded as the primary emission wavelength that should be developed in III-nitride LEDs for new applications such as UV curing, purification, and biological and medical applications.

To pursue alternative next-generation TCEs, various attempts have been made using new types of functional and nanoscale materials, such as carbon nanotubes^27^,^28^,^29^,^30^, graphene^31^,^32^,^33^,^34^,^35^,^36^,^37^,^38^,^39^, conductive polymers^40^, and silver nanowires (AgNWs)^41^,^42^,^43^,^44^,^45^,^46^,^47^. These approaches have been more actively applied in the development of organic semiconductors than in inorganic III-nitride semiconductors. This is presumably due to the larger technical barriers (i.e., the difficulties in forming an ohmic contact to *p*-type GaN (*p*-GaN) and incompatibility in photolithographic patterning with new materials) and a lesser need for flexibility. Furthermore, the performance of LEDs fabricated with new electrodes has not been sufficient to replace ITO electrodes. For example, LEDs fabricated with graphene TCEs have suffered from complex process flow (required for the growth and transfer of graphene layers), large forward voltage (caused by the poor graphene ohmic contact to the *p*-layer and by the high *R*_sh_ of monolayer graphene), poor reproducibility associated with atomic-dimension processes, and reliability issues associated with the reaction of graphene with ambient air^33^,^34^,^35^,^36^,^37^,^38^,^39^.

Recently, our groups demonstrated that solution-processed, high-quality AgNW electrodes can be successfully used in organic solar cells as a result of the guaranteed properties of low *R*_sh_ (~10 Ω/sq), high *T*_op_ (~95% at 550 nm), and excellent flexibility^45^,^46^,^47^. Furthermore, the process is quite simple and cheap relative to processes such as the use of graphene and/or ITO, suggesting that solution-processed AgNWs are a promising potential candidate material for use in next-generation TCEs of III-nitride LEDs. However, in-depth comprehensive studies on this subject are lacking. Here, we demonstrate III-nitride LEDs fabricated with AgNW TCEs (referred to hereinafter as AgNW-LEDs). First, the electrical, optical, and structural properties of solution-processed (or spin-coated) AgNWs were investigated as a function of the annealing temperature. The optimized AgNW films that were approximately 70 nm thick showed excellent *T*_op_ at the industrially important visible and deep UV wavelengths of 450 and 260 nm, respectively. The feasibility of ohmic contact is a prerequisite for the application of AgNWs to III-nitride LEDs because the *p*-GaN layer’s large work function (associated with the wide GaN bandgap, *E*_g_ = 3.4 eV) and low carrier densities create a large Schottky barrier at the contact/*p*-GaN interface^3^. To investigate whetherthe AgNWs can produce ohmic contacts to *p*-GaN, we employed transmission line model (TLM) methods^48^ using specifically designed patterns. Interestingly, the AgNW contact on the *p*-layer produced near ohmic behaviour and exhibited significant bias-voltage dependence, leading to a significantly enhanced ohmic contact under high bias voltage or high current density. LEDs fabricated with AgNW TCEs showed superior electrical and optical performance to the reference LEDs, which was attributed to the combined effects of better current spreading (due to a low *R*_sh_ value), enhanced ohmic behaviour under high bias voltages, and an excellent *T*_op_.

## Results

### Electrical and optical properties of AgNWs

**Figure 1:**
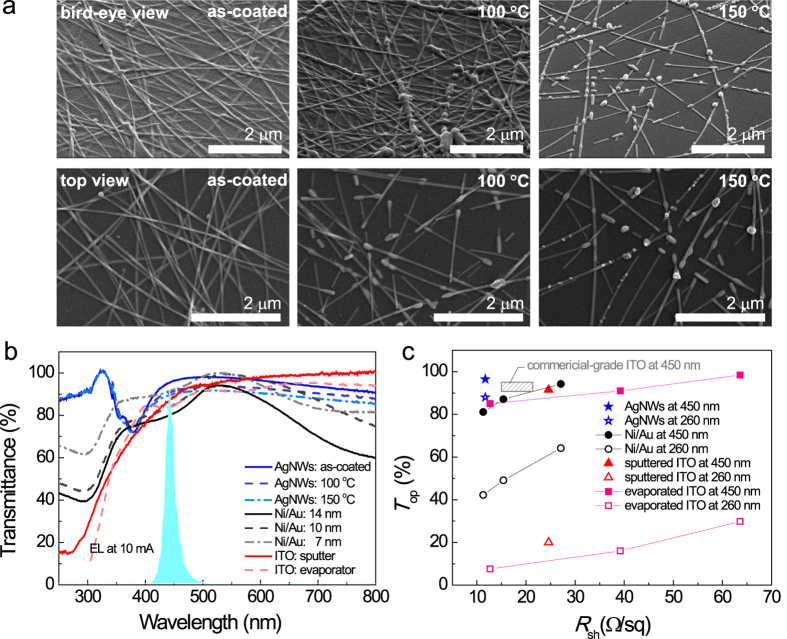
(**a**) SEM top views and bird’s-eye views of the AgNW films coated on a sapphire substrate before and after thermal annealing at 100 and 150 °C. (**b**) Optical specular transmittance spectra and (**c**) optical transmittance versus *R*_sh_ plots of the as-coated and annealed AgNW films; oxidized Ni/Au films with overall thicknesses of 7, 10, and 14_ nm_; and 100-nm-thick ITO films.

Figure 1a shows scanning electron microscopy (SEM) top views and bird’s-eye views of AgNW films coated on a sapphire substrate before and after thermal annealing at 100 and 150 °C in an N_2_ ambient. The as-coated sample showed a typical squashed arrangement of AgNWs with an average diameter of ~30 nm and average length of a few tens of micrometres, which is consistent with the literature^45^,^46^,^47^. After thermal annealing, AgNWs were agglomerated and disconnected, indicating that the as-coated condition was the best in terms of *T*_op_ and electrical conductivity.

Figure 1b shows the optical specular transmittance spectra of the as-coated and annealed AgNW films; for comparison, the transmittance spectra of reference TCEs, including the oxidized Ni/Au with a different overall thickness of 7, 10, and 14 nm, *e*-beam evaporated 400 nm-thick ITO films, and sputtered 100 nm-thick ITO films, are also plotted. As expected, the as-coated AgNW sample produced the highest *T*_op_ among the AgNW samples. Furthermore, this AgNW sample had high *T*_op_ across the entire wavelength region tested, except at 380 nm, which is associated with the transverse plasmon mode of AgNWs^42^. The *T*_op_ of the ITO films was high in the visible wavelength region but began to drop significantly at wavelengths below 400 nm owing to ITO’s band-to-band absorption. The *T*_op_ of the oxidized Ni/Au samples showed significant negative dependence on their thickness, i.e., the thicker a Ni/Au film was, the lower its *T*_op_ was. In addition, the Ni/Au samples showed relatively poor *T*_op_ at wavelengths below approximately 350 nm and above 600 nm, which is directly associated with the optical absorption spectra of Au films^49^. In this study, the primary wavelength of interest was 450 nm because the developed TCEs were applied to commercial LED wafers with blue emission (as plotted in Fig. 1b), where the EL spectrum of the fabricated LEDs was acquired at 10 mA. In addition, the *T*_op_ at the deep UV wavelength of 260 nm (*T*_op@260nm_) was monitored in the study because this wavelength is of particular interest to the improvement of the extraction efficiency for deep UV LEDs^50^.

To determine the best TCEs, *R*_sh_ should also be taken into consideration. Figure 1c summarizes the *T*_op_ versus *R*_sh_ plots of the as-coated AgNWs, oxidized Ni/Au films with the overall thicknesses of 7, 10, and 14 nm, and ITO films. Evidently, AgNW films showed the best figures of merit: *T*_op@260 nm_ = 87.8%, *T*_op@450 nm_ = 96.5%, and *R*_sh_ = 11.7 Ω/sq. Meanwhile, the ITO film had relatively poor electrical and optical properties, i.e., *T*_op@260 nm_ = 29.8/16.0/7.6%, *T*_op@450 nm_ = 98.4/91.0/85.0%, and *R*_sh_ = 63.6/39.2/12.7 Ω/sq for the *e*-beam evaporated 200/300/400_ nm_-thick ITO films and *T*_op@260 nm_ = 20.0%, *T*_op@450 nm_ = 91.5%, and *R*_sh_ = 24.6 Ω/sq for the sputtered ITO films. Notably, the *R*_sh_ of Ni/Au films (as well as their *T*_op_) depended strongly on their layer thickness: *R*_sh_ = 27.1/15.4/11.3 Ω/sq and *T*_op@450 nm_ = 94.2/87.1/81.1% for the overall thicknesses of 7, 10, and 14 nm. These results indicate that the as-coated AgNW films are promising as TCEs for enhancing the performance characteristics of LEDs for blue and deep UV applications. In this study, blue-emitting LEDs were adopted to verify the usability of AgNW TCEs because they are regarded as the standard platform in terms of industrial mass production. In addition, Ni/Au films were used as the reference electrodes because their *T*_op_ and *R*_sh_ values were comparable to those of ITO films (although the properties were slightly poorer than those of commercial-grade ITO) and the ohmic contact to *p*-GaN was very good, as will be discussed below. The optical and electrical properties of the Ni/Au and AgNW electrodes are presented in Table 1.

### Contact properties of AgNWs

**Figure 2:**
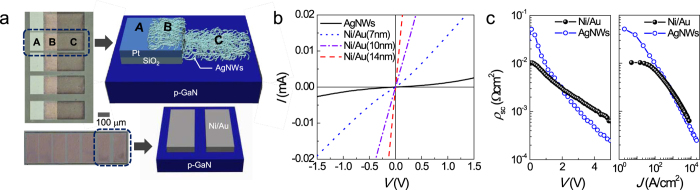
(**a**) Schematic cross-section and optical microscopic top view of the AgNW TLM pads and Ni/Au reference TLM pads. (**b**) *I–V* curves of the AgNW and the 7-, 10-, and 14-nm-thick Ni/Au TLM pads, as measured from adjacent contact pads with the spacing of 10 μm. (**c**) *ρ*_sc_ of the AgNW and the14-nm-thick Ni/Au electrodes versus the bias voltage and current density.

To fabricate reliable LEDs with AgNW electrodes, it is crucial to form AgNW ohmic contact to *p*-GaN. To investigate the contact property, the specifically designed TLM patterns shown in Fig. 2a were used. In this structure, AgNWs overlie the bottom *p*-GaN surface and SiO_2_/Pt probing pads, enabling measurement of the pure AgNW contact on the *p*-layer by probing the SiO_2_/Pt pads. Figure 2a shows a schematic cross-section and optical microscopic top view of the reference electrode with a 14nm-thick oxidized Ni/Au layer. Figure 2b shows the current–voltage (*I–V*) curves of the AgNWs and 7, 10, and 14 nm-thick Ni/Au TLM pads, as measured from adjacent contact pads with spacing of 10 μm. It is evident that the *I–V* curves of the Ni/Au electrodes are much steeper than that of the AgNW electrode. In addition, both reference Ni/Au electrodes and the AgNW electrode produced nearly linear *I–V* curves (for the 10- and 14-nm-thick Ni/Au) and very slightly nonlinear *I–V* curves (for the 7-nm-thick Ni/Au and AgNWs), indicating the formation of ohmic contact. For example, the specific contact resistance (*ρ*_sc_) estimated based on the measured electrical resistance (*R*) at 1.0 V, i.e., *R* = *V*/*I* = 1.0 V/*I*, was as low as 2.2 × 10^−3^ and 3.5 × 10^−3^ Ωcm^2^ for the 14 nm-thick Ni/Au electrodes and AgNW electrodes, respectively. However, this prediction might be inaccurate because of the nonlinearity of the *I–V* curves, as will be discussed in detail below.

One of the major concerns in Fig. 2b is the different amount of current flow, which is particularly pronounced in AgNW electrodes. The possible origins for the significantly reduced *I–V* slopes can be inferred based on the thickness dependence of the reference Ni/Au electrodes: the higher the sheet resistance is (i.e., the thinner the electrode is), the worse the *I–V* slope is. Based on this tendency, the lowest *I–V* slope of the AgNW electrode might be due to the degraded *R*_sh_ of the AgNWs. Indeed, this explanation is plausible considering that the electrical conductivity of the AgNWs might be degraded due to the use of an overlying structure of AgNWs on the bottom *p*-GaN surface and the Pt pads; specifically, the AgNWs at the boundary region of the *p*-GaN window and the 30 nm-thick probing pads might be disconnected as a result of the height difference. This possibility was confirmed by analysing the probing-position-dependent *I–V* curves of AgNW films (Supplementary Fig. 1). For example, with a constant probing distance of approximately 80 μm, the *I–V* curve obtained by probing only the C region was steeper than that obtained by probing both the B and C regions. Another possible origin, which appears to be a much more dominant factor, is the difference of contact area. For example, whereas the reference electrodes cover the entire *p*-GaN surface defined by the photographic patterning, the AgNWs occupy only a small fraction of the defined area owing to their zero- or one-dimensional NW contacts. Consistently, the measured *R*_sh_ values of the *p*-GaN layer determined from the AgNWs and 14-nm-thick Ni/Au samples according to the TLM theory were 2.9 × 10^7^ and 1.0 × 10^5^ Ω/sq, respectively. Despite the use of the same wafer, the two orders of magnitude higher *p*-layer resistance obtained from the AgNW sample is indicative of reduced contact area.

One thing we have to remind is the nonlinearity of the *I–V* curves, particularly pronounced for the AgNW electrodes. Nonlinearity was even observed for the 14nm-thick Ni/Au electrodes when the *V–I* curve was plotted in the voltage range from −5 to +5 V (Supplementary Fig. 2). This suggests that a corrected method should be taken into consideration to evaluate *ρ*_sc_ accurately for our samples because the conventional TLM theory is only valid when the *I–V* curve is perfectly linear or when perfect ohmic contact is formed. Recently, Piotrzkowski *et al.*^51^, while investigating corrected *ρ*_sc_ values when the *I–V* curve was nonlinear, proposed a universal method to obtain bias-voltage-dependent or injected-current-density-dependent *ρ*_sc_ values according to


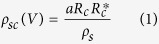


where *a* is the width of the TLM pattern and *R*_c_ = *V*/*I*_0_ and *R*_c_^*^ = d*V*/d*I*_0_ are the common and differential front resistances of single planar contact in TLM structure, respectively (Supplementary Fig. 3)^51^. Using this model, reasonable *ρ*_sc_ values were obtained as a function of the bias voltage and current density (*J*), as shown in Fig. 2c. Note that the *ρ*_sc_ values of the AgNW electrodes are more significantly dependent on the bias voltage and current density than those of the Ni/Au electrodes. As a result, whereas the *ρ*_sc_ value of the AgNW electrodes was higher than that of the reference 14 nm-thick Ni/Au electrodes at *V* < 1.95 V, those of AgNW electrodes became much lower at *V* > 1.95 V, e.g., *ρ*_sc_ = 1.2 × 10^−2^ and 6.8 × 10^−3^ Ωcm^2^ at *V* = 1.0 V and 2.5 × 10^−4^ and 6.3 × 10^−4^ at *V* = 5.0 V for the AgNW and Ni/Au electrodes, respectively. A possible cause of the stronger field dependence of the AgNW electrodes than the Ni/Au electrodes is the zero- or one-dimensional NW contact. For example, higher bias induces more current to flow through a small cross section, resulting in Joule heating. The temperature rise in such a localized area leads to a *ρ*_sc_ reduction owing to enhanced thermionic emission^52^ and/or improved adhesion between AgNWs and *p*-GaN via interfacial reaction, causing more current to flow and accelerated Joule heating. Notably, this process would be self-reinforcing, leading to a self-accelerating thermal process. Indeed, it is instructive to recall how the observed field-enhanced NW ohmic contact affects the electrical and optical performance characteristics of AgNW-LEDs, particularly at higher bias voltages.

### Performance characteristics of AgNW-LEDs

**Figure 3:**
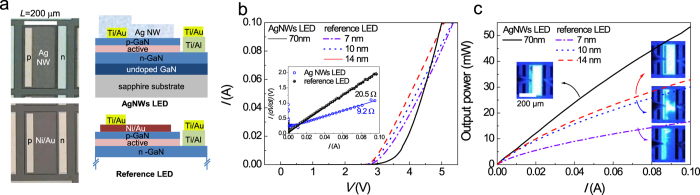
(**a**) Optical microscopic top views and schematic cross-sectional diagrams of AgNW-LEDs and reference LEDs. (**b**) *I–V* curves of the AgNW-LEDs and reference LEDs. (b, inset) *I*(*dV*/*dI*) versus *I* plots. (**c**) Optical output power versus injection current for AgNW-LEDs and reference LEDs. (c, inset) EL images taken at 2 mA.

Figure 3a shows the optical microscopic top views and schematic cross-sectional diagrams of AgNW-LEDs and reference LEDs fabricated with Ni/Au electrodes. In this figure, it is shown that the AgNWs were formed on the bottom *p*-GaN surface and Au probing pads. In contrast, the Ni/Au electrode was first deposited on the *p*-GaN, on which the probing pad was formed. The length (*L*) and width (*W*) of the mesa were 200 and 500 μm, respectively.

Figure 3b shows the *I–V* curves of AgNW-LEDs and reference LEDs. As expected from the TLM results, the forward *I–V* curves of the AgNW-LEDs showed distinctive behaviour compared to the reference LEDs. For example, the current flow of AgNW-LEDs was small in the low voltage range but began to rapidly increase with increasing bias voltage, particularly above ~4.0 V. Accordingly, the series resistance of the devices (*R*_s_) extracted according to the relation *I*(*dV*/*dI*) = *IR*_*s*_ + *nkT*/*q*, where *n* is the ideality factor, *k* is the Boltzmann constant, and *T* is the absolute temperature, was 9.2 Ω for the AgNW-LEDs and 20.5 Ω for reference LEDs fabricated with 14 nm-thick Ni/Au electrodes (Fig. 3b inset). This finding is quite interesting because, despite the expected smaller effective active area, the AgNW-LEDs showed a 55% lower *R*_s_ value compared to the reference LEDs. As discussed above, a possible mechanism for this finding might be field-enhanced carrier transport through the AgNW/*p*-GaN interface, as observed in Fig. 2c.

Figure 3c shows the optical output power versus injection-current curves of the LEDs. Notably, the AgNW-LEDs had significantly higher optical output power than the reference LEDs. For example, the optical output powers measured at 80 mA were 14.4, 25.8, 28.3, and 44.3 mW for the LEDs fabricated with 7, 10, and 14 nm-thick Ni/Au electrodes and an AgNW electrode, respectively. In the reference LEDs, the significant drop of optical output power with decreasing Ni/Au thickness (despite its higher optical transmittance) was attributed to the degraded current spreading associated with increased *R*_sh_ values, i.e., operation in the current-spreading limited regime^53^, as shown in the electroluminescence (EL) images of Fig. 3c. Specifically, a significant current crowding was observed near the *p*-probing pads; current crowding is regarded to originate from the poor electrical conductivity of the Ni/Au electrodes rather than that of the *n*-GaN layer (13.9 Ω/sq)^53^ Here, the *R*_sh_ value of the *n*-layer was estimated from an *n*-contact using the TLM method. Therefore, LEDs fabricated with 14 nm-thick Ni/Au electrodes are hereafter referred to as the true reference LEDs because they performed the best. Once more, note that the AgNW-LEDs showed optical output power 56.5% higher than that of the reference LEDs.

## Discussion

**Figure 4:**
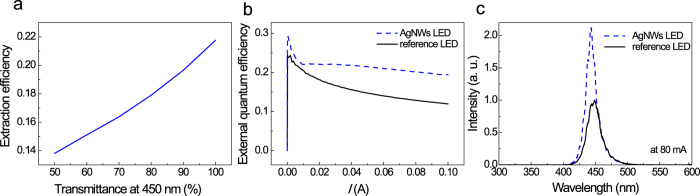
(**a**) Calculated extraction efficiency of LEDs versus TCE optical transmittance. (**b**) External quantum efficiency versus current curves for AgNW-LEDs and reference LEDs. (**c**) EL spectra of AgNW-LEDs and reference LEDs taken at the injection current of 80 mA.

The significantly improved optical output power of AgNW-LEDs is essentially due to the AgNW electrode having a higher *T*_op_ (96.5%) than the Ni/Au electrode (81.1%). However, it is questionable whether the 18% increase in the *T*_op_ can lead to the 56.5% higher optical output power observed. To investigate this question, an optical ray-tracing simulation was performed using the real device configuration and optical constants (Fig. 4a). Details of the optical ray-tracing simulations can be found elsewhere^54^,^55^. The calculations showed that the 18% increase in the *T*_op_ could lead to ~17% higher optical output power, indicating that another factor also contributed to the enhanced output power of the AgNW-LEDs.

The efficiency droop, that is, the loss of external quantum efficiency at high injection current, was also found to significantly decrease in the AgNW-LEDs, as shown in Fig. 4b. For example, the efficiency droop values measured from the maximum peak intensity to those measured at 100 mA were 34.3 and 50.8% for the AgNW-LEDs and reference LEDs, respectively. Several studies have reported that the efficiency droop originates variously from crystallographic defects^56^,^57^,^58^,^59^ such as point defects and threading dislocations, Auger recombination^60^,^61^,^62^, electron leakage^63^,^64^ (caused by limited hole-injection efficiency^65^, ineffective electron blocking layers^66^, and incomplete carrier capture by quantum wells)^67^, current crowding^68^, and heating effects^69^. Among these possible origins, the effect of defects can be neglected because the same LED wafers were used in this study. In addition, the two representative origins of Auger recombination and electron leakage, which respond sensitively to the carrier density injected into the active region, can also be ignored because the efficiency droop of the AgNW-LEDs was greatly alleviated despite their much higher expected current density associated with the reduced active area. A reasonable explanation for the reduced efficiency droop, therefore, might be a reduction in the heating effect and/or improved current spreading, which would also be responsible for the improved optical output power. Indeed, the reduced heating effect in the AgNW-LEDs is acceptable according to the lower *R*_s_ value (9.2 Ω) and Joule's first law, i.e., *Q*∝*I*^2^*R*_s_, where *Q* is the generated heat. Accordingly, the EL peak position of the AgNW-LEDs taken at the injection current of 80 mA was blue-shifted by 4 nm compared to the reference LEDs (Fig. 4c). This is consistent with the reduced heating effect explanation, considering that a temperature increase results in a red shift owing to bandgap shrinkage^70^. The blue shift of the EL spectra might also be due to the increased band filling in the AgNW-LEDs, which is associated with a higher density of injected current^7^.

**Figure 5:**
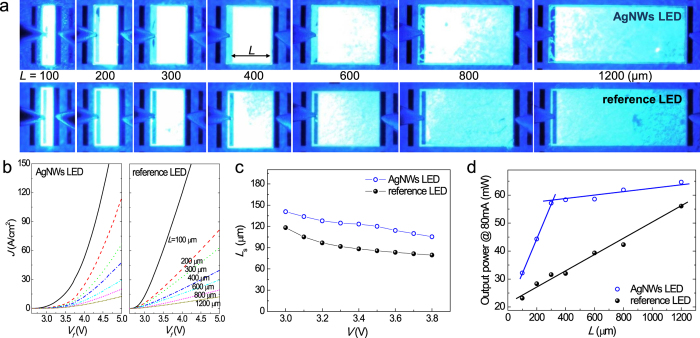
(**a**) LED test structures with various lateral mesa lengths ranging from *L* = 100 to *L* = 1200 μm; images were obtained during operation at an injection current of 5 mA. (**b**) *J–V* curves of AgNW-LEDs and reference LEDs of various *L*. (**c**) *L*_s_ values obtained for both LED types as a function of the bias voltage. (**d**) Area dependence of the optical output power of both LED types.

To investigate another hypothesis that the current spreading is better in the AgNW-LEDs, we fabricated test structures with various lateral mesa lengths (*L*) ranging from 100 to 1200 μm and collected EL images while operating the test structures at an injection current of 5 mA (Fig. 5a). The current is spread more uniformly in the AgNW-LEDs; specifically, the current spread perfectly for AgNW-LED structures with *L* = 400 μm, and for reference LED structures of *L* = 200–300 μm. To obtain the current spreading length (*L*_s_), defined as the length over which the current density drops to the 1/*e* value at the mesa edge, the current densities *J* of both LEDs were plotted as a function of the forward voltage and *L*, as shown in Fig. 5b. Using the experimental *J−L* characteristics re-plotted as a function of the bias voltage (see the Supplementary Fig. 3), the *L*_s_ was estimated by theoretical fitting of the experimental *J−L* data using the following equation:


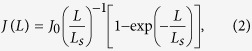


where *J*_*0*_ is the current density at the mesa edge. The detailed theoretical background and the procedure to obtain the *L*_s_ can be found elsewhere^71^,^72^,^73^. Figure 5c shows the *L*_s_ values obtained as a function of the bias voltage. It is evident that the *L*_s_ values of the AgNW-LEDs were longer than those of the reference LEDs by approximately 30 μm, which is consistent with the EL images. Therefore, the enhanced optical output power and reduced efficiency droop of the AgNW-LEDs can also be due to the improved current spreading, which may contribute to the reduced *R*_s_ value. However, the *L*_s_ values (approximately 120 μm) are likely to be underestimated. This is attributed to the lack of an exact theoretical model to describe our LED samples. Eq. (2) is only valid when the vertical *p*-layer resistances, including the *p*-contact and the *p*-GaN, are negligibly small^71^,^72^,^73^, whereas that of our samples, particularly for the AgNW-LEDs, is not small enough to ignore and, more importantly, has a significant bias voltage dependence.

**Figure 6:**
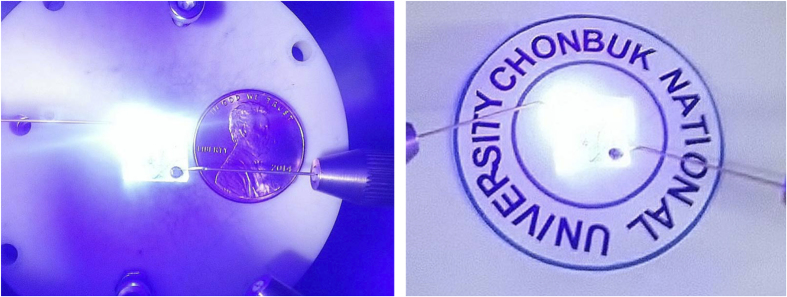
EL images of 1 cm × 1 cm large-area LEDs taken at 50 mA.

The better electrical and optical performance observed for AgNW-LEDs and their longer *L*_s_ values suggest that AgNWs can be used as next-generation TCEs in large-area devices. The suitability of AgNW electrodes for large-area devices is further confirmed from their area dependence of optical output power, as shown in Fig. 5d. At a constant current injection of 80 mA, the optical output power increased with increasing *L* (or device area), which is essentially due to the reduced efficiency droop associated with the decreased current density. Interestingly, the optical output powers of AgNW-LEDs increased rapidly up to a high level at *L* ≤ 300 μm and changed insignificantly at *L* ≥ 300 μm, whereas those of the reference LEDs gradually increased with increasing *L* up to 1200 μm. This result suggests that the AgNW TCEs are suitable for use in large-area devices owing to their higher optical output power and less area-dependent output efficiency, as demonstrated in Fig. 6. Figure 6 shows the EL images of 1 cm × 1 cm large-area LEDs fabricated with AgNW TCEs (at 50 mA). The devices were simply fabricated using a quick-test structure^74^ and indium as both the *n*- and *p*-contacts. The LEDs showed bright light emission across the entire active region, suggesting that the large-area LEDs could be fabricated using efficient AgNW spreading contact.

**Figure 7:**
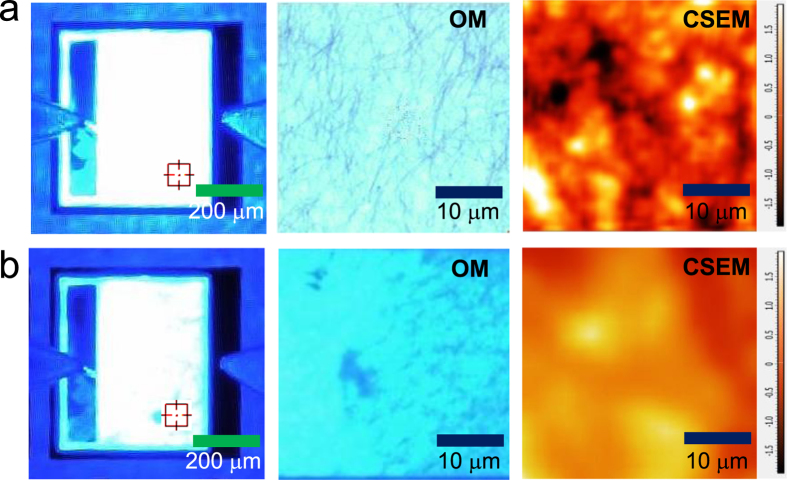
Magnified EL and CSEM images of (a) AgNW-LEDs and (b) reference LEDs taken at 10 mA.

So far, the field-enhanced NW ohmic contact (making a strong localized current flow underneath the NW contact), the improved current spreading, and excellent optical transmittance were found to cause the enhanced performance characteristics of AgNW-LEDs. Although improved current spreading was observed in the EL images, the images do not constitute evidence of localized current flow underneath the AgNW contact. It is also questionable whether the localized current flow through the NW contact could be large enough to explain the improved electrical properties. For this purpose, spatially-resolved EL images were observed using confocal scanning electroluminescence microscopy (CSEM)^75^,^76^ (Fig. 7). For this measurement, the standard LED structure with surrounding *n*-electrodes was used (Supplementary Fig. 4). The dimensions of the mesa were 400 μm × 500 μm. EL images taken at an injection current of 10 mA revealed that the current spreading of AgNW-LEDs (Fig. 7a) was more uniform than that of the reference LEDs (Fig. 7b), even though the surrounding *n*-electrodes were employed. The magnified EL images from the areas indicated by targets in Fig. 7 showed brighter and more uniform light emission (along with squashed NWs) in the AgNW-LEDs than in the reference LEDs. At a similar position with identical magnification, high-resolution CSEM showed non-uniform light emission with a few very bright localized spots in the AgNW-LEDs, supporting our arguments. CSEM showed uniform emission in the reference LEDs, in which the broad emission inhomogeneity was associated with the non-uniform current spreading. In Fig. 7a, more specifically, the localized brighter emission had shapes of short dots and lines, which predicted the physical shape of the AgNW/*p*-GaN contact. In the CSEM images, the light emission near the bright localized spots was not much less than, or was even comparable to, the average brightness of the reference LEDs, indicating that the carrier transport around the AgNW contact was also active. However, a very dark area was also observed, which should be avoided to obtain better performance of AgNW-LEDs. Specifically, process optimization to achieve a more uniform contact between the NWs and the *p*-layer, which may be obtained using an intermediate contact layer, will be challenging.

## Methods

### Formation of AgNW films and reference electrodes

To form AgNW films, an as-received dispersion containing AgNWs (CambriosClearOhm Ink) was sonicated for 300 s, shaken well, and then spin coated on a pre-cleaned sapphire substrate for 40 s at 800 rpm^45^,^46^,^47^. The AgNW films were evaluated before and after a rapid thermal annealing at 100 and 150 °C for 1 min in N_2_ ambient. For a comparative study as a reference electrode, Ni/Au bilayers with thicknesses of 3.5/3.5, 5/5, and 7/7 nm were deposited on the sapphire substrate using an *e*-beam evaporator, followed by rapid-thermal annealing at 550 °C for 1 min in an O_2_ ambient^77^,^78^. Additionally, ITO films were deposited on the sapphire substrate using an *e*-beam evaporator or a radio-frequency (rf) magnetron sputtering system with an rf power of 100 W, deposition time of 6 min, temperature of 500 ºC, Ar:O2 gas ratio of 10:0, and working pressure of 10 mTorr, followed by rapid-thermal annealing at 550 °C for 1 min in oxygen ambient. The spin-coated AgNW films were analysed by SEM, revealing that their overall thickness was approximately 70 nm. The optical transmittance and Rsh of the AgNW films, Ni/Au, and ITO layers were measured using a UV/VIS spectrometer (V-670EX) and a four-point probe system (CMT-SR1000N).

### Evaluation of contact properties

To investigate the contact properties, TLM patterns specifically designed to measure the AgNW contact on *p*-GaN by probing adjacent SiO_2_/Pt pads were fabricated, as shown in Fig. 2a. For example, 20 nm-thick SiO_2_ films were deposited on the top *p*-layer of an LED wafer by an *e*-beam evaporator; subsequently, the SiO_2_ layer was selectively wet etched (using buffered oxide etchants) to expose the *p*-layer surface for AgNW contact. Then, a 10 nm-thick Pt pad was selectively deposited on the edge of the exposed *p*-layers, followed by selective coating of the AgNWs onto the exposed *p*-layer and the Pt pads simultaneously. The photolithographic lift-off technique was used for the selective deposition of the Pt pad and AgNW contact. For a comparative study, an oxidized Ni/Au electrode was also evaluated using the standard TLM patterns. For the AgNW electrodes, the TLM pattern included 150 × 200 μm contact pads and spacings of 10, 20, 40, and 60 μm; for the Ni/Au electrodes, the TLM pattern included 100 × 200 μm contact pads and spacings of 5, 10, 15, 20, 25, and 30 μm. The reason for the use of the larger pattern size for AgNWs was the poorer patterning accuracy of AgNWs than of Ni/Au. The electrical properties of the contacts were evaluated using a parameter analyser (HP4156A).

### Demonstration of AgNW-LEDs

To fabricate LEDs with AgNW TCEs, the same method that was used to form the AgNW TLM pattern was utilized (Supplementary Fig. 5). Specifically, the rectangular mesa was defined by dry etching to a thickness of ~1.0 μm to expose the *n*-layer using an inductively-coupled plasma reactive ion etching system on which a Ti/Al/Ni/Au (30/70/30/70 nm) layer was deposited as an *n*-electrode by an *e*-beam evaporator. Rapid thermal annealing was performed at 550°C for 1 min in ambient N_2_ to form an *n*-type ohmic contact. To form AgNW TCEs on the *p*-layer, a Ti/Au (20 nm/10 nm) probing pad was formed on the mesa, followed by selective AgNW coating on the exposed *p*-layer and Ti/Au probing pads by means of a lift-off technique. The spin-coating process was performed in the last process step to minimize the possible contamination of AgNWs by additional photolithographic processing. To implement our study, commercially available LEDs wafers were used; these were grown on *c*-plane sapphire substrates by a metalorganic chemical vapour deposition. The structure of the LEDs comprised 2.0 μm of undoped GaN, 3.5 μm of *n*-GaN, 5-period GaN/InGaN multiple quantum well (MQW) active regions with 450 nm-emission, a 0.024 μm *p*-AlGaN electron blocking layer, and a 0.14 μm *p*-GaN layer. The fabricated LEDs were evaluated using a parameter analyser connected to a photodiode (UV-818) and an optical spectrometer (Ocean Optics-USB2000+). To investigate the spatially-resolved electroluminescence (EL) images, CSEM^75^,^76^ was employed.

## Additional Information

**How to cite this article**: Oh, M. *et al.* Silver Nanowire Transparent Conductive Electrodes for High-Efficiency III-Nitride Light-Emitting Diodes. *Sci. Rep.* **5**, 13483; doi: 10.1038/srep13483 (2015).

## Supplementary Material

Supplementary Information

## Figures and Tables

**Table 1:** The optical and electrical properties of the Ni/Au and AgNW electrodes.

	**TCE properties**			**Conventional TLM method**		**Universal TLM method**	
	***T*_op@260 nm_(%)**	***T*_op@450 nm_(%)**	***R*_sh___TCE_(Ω/sq)**	***ρ*_sc_ (Ω cm^2^)**	***R*_sh*p*-GaN_(Ω/sq)**	***ρ*_sc@1 V_(Ωcm**^2^)	***ρ*_sc@5 V_(Ωcm**^2^)
Ni/Au (7 nm)	64.2	94.2	27.1				
Ni/Au (10 nm)	49.1	87.1	15.4				
Ni/Au (14 nm)	42.2	81.1	11.3	2.2 × 10^−3^	1.0 × 10^5^	6.8 × 10^−3^	6.3 × 10^−4^
AgNWs	87.8	96.5	11.7	3.5 × 10^−3^	2.9 × 10^7^	1.2 × 10^−2^	2.5 × 10^−4^
