# Supplementary material for: Silver Nanowire Transparent Conductive Electrodes for High-Efficiency III-Nitride Light-Emitting Diodes
Source: Sci Rep. 2015 Sep 3;5:13483. doi: 10.1038/srep13483 (PMC4558582; doi:10.1038/srep13483)
Supplement: Supplementary Information [file srep13483-s1.pdf]

Silver Nanowire Transparent Conductive Electrodes for High-Efficiency III-Nitride Light-Emitting Diodes

Munsik Oh[1], Won-Yong Jin[2], Hyeon Jun Jeong[3], Mun Seok Jeong[3], Jae-Wook Kang[2], and Hyunsoo Kim[1]

[1]*School of Semiconductor and Chemical Engineering, Semiconductor Physics Research Center, Chonbuk National University, Jeonju 561-756, Republic of Korea.* [2]*School of Flexible and Printable Electronics, Polymer Materials Fusion Research Center, Chonbuk National University, Jeonju 561-756, Republic of Korea.* [3]*Center of Integrated Nanostructure Physics, Institute for Basic Science, Sungkyunkwan University, Suwon 440-746, Republic of Korea. Correspondence and requests for materials should be addressed to J.-W. K. (email:* jwkang@jbnu.ac.kr) *and H. K. (email:* hskim7@jbnu.ac.kr).

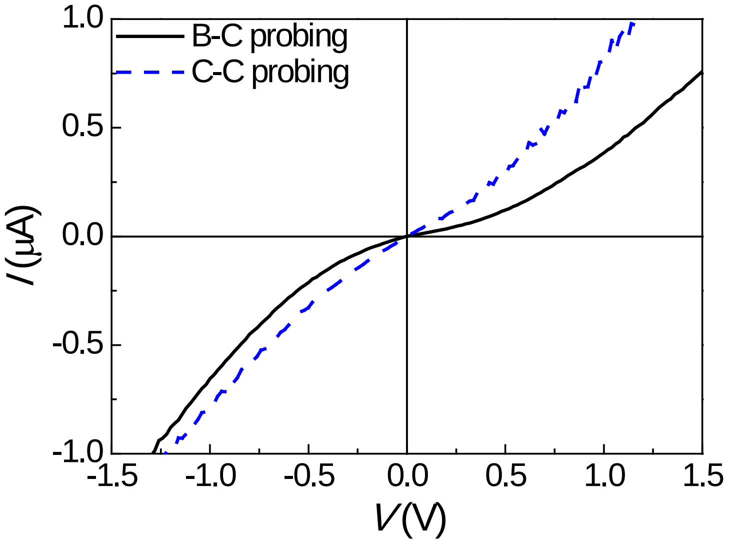

Supplementary Figure 1 Probing-position-dependent *I–V* curves of AgNW films.

With a constant probing distance of approximately 80 μm, the *I–V* curve obtained by probing only the C region was steeper than that obtained by probing both the B and C regions. This indicates that the electrical conductivity of the AgNWs might be degraded due to the use of an overlying structure, where the AgNWs at the boundary region of the *p*-GaN window and the 30 nm-thick probing pads might be disconnected due to the height difference.

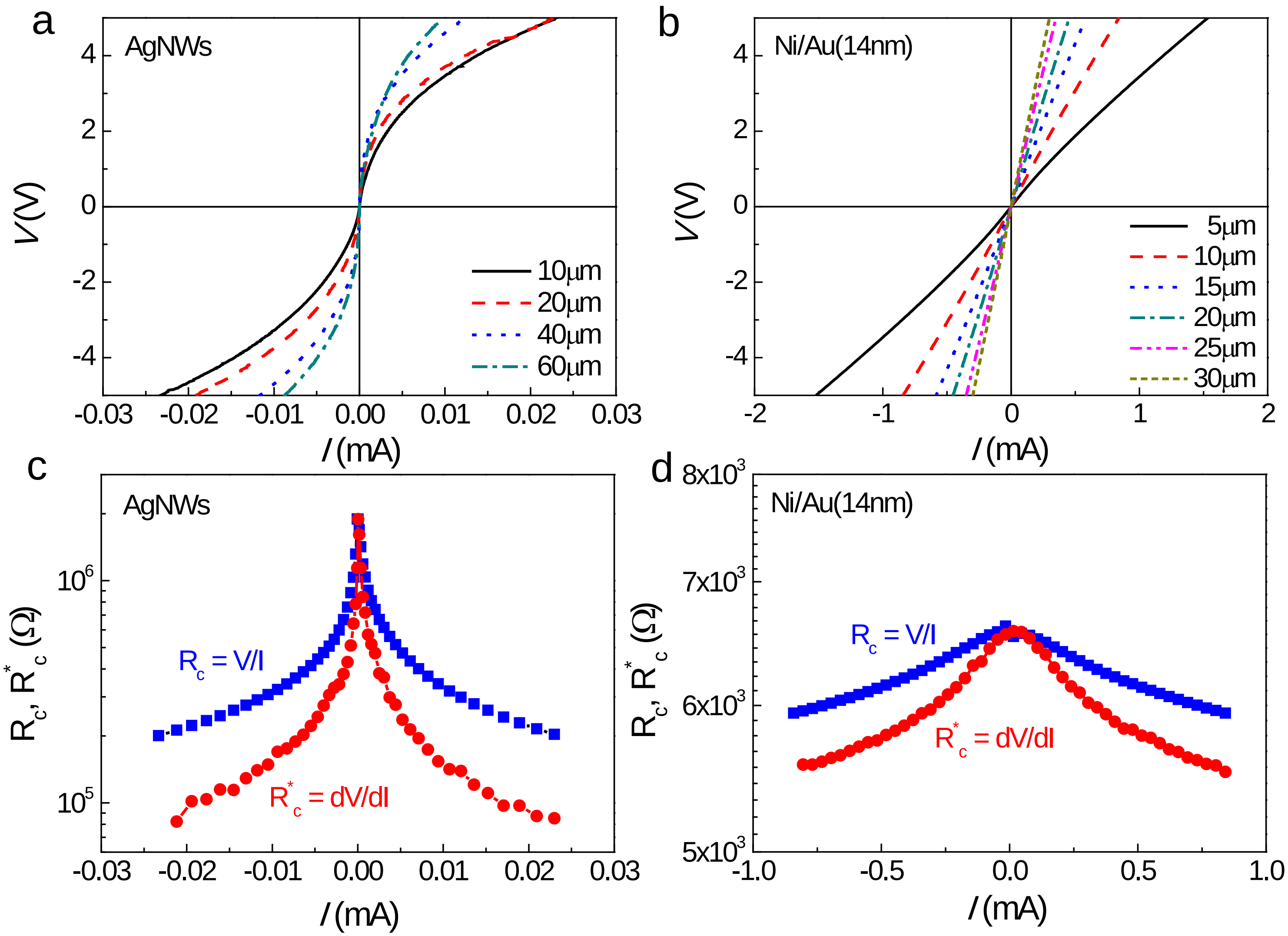

Supplementary Figure 2 *V-I* curves and R_c, R_c^*-*I* curves of AgNWs and Ni/Au contacts

Supplementary Figure 2a and 2b show the *V-I* curves of AgNWs and Ni/Au contacts, exhibiting a non-linearity at zero currents. Supplementary Figure 2c and 2d show the R_c and R_c^* plots of AgNWs and Ni/Au contacts, as calculated from the relation of $R_c=V/I_0$ and $R_c^*=\mathrm{d}V/\mathrm{d}I_0$, where I_0 is the total current flowing from metal pad to sample layer. Using the obtained R_c and R_c^* values, the bias-voltage-dependent contact resistance ρ_{sc} (V) can be obtained according to $\rho_{sc}(V) = aR_cR_c^*/\rho_s$, where a is the width of TLM pattern and ρ_s is the specific resistivity of semiconductor.

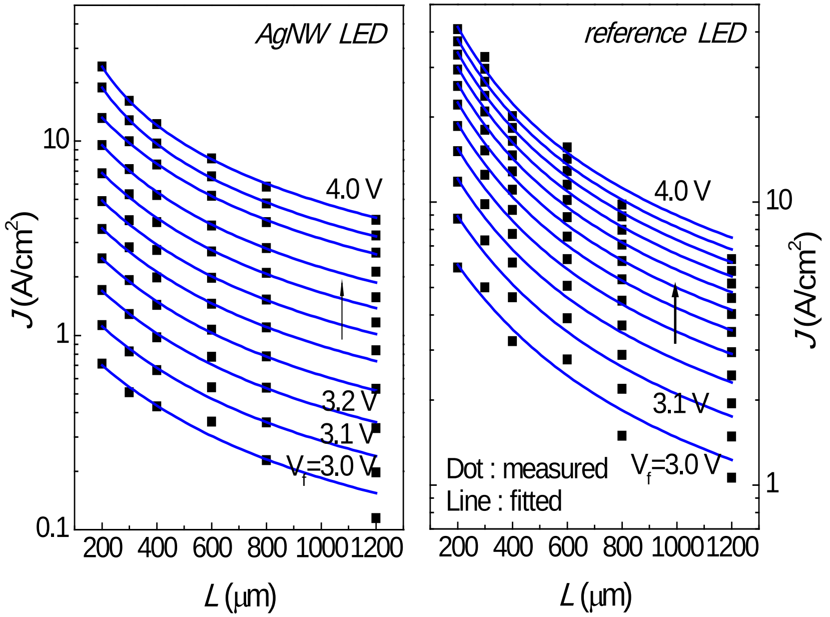

Supplementary Figure 3 *J–L* characteristics of both LEDs as a function of the bias voltage

The current spreading length (L_s) can be obtained by theoretical fitting (shown as blue lines in above figures) of the experimental *J–L* data (dots) using the following equation of $J(L)=J_0(L/L_s)-1[1-\exp(-L/L_s)]$, where J_0 is the current density at the mesa edge.

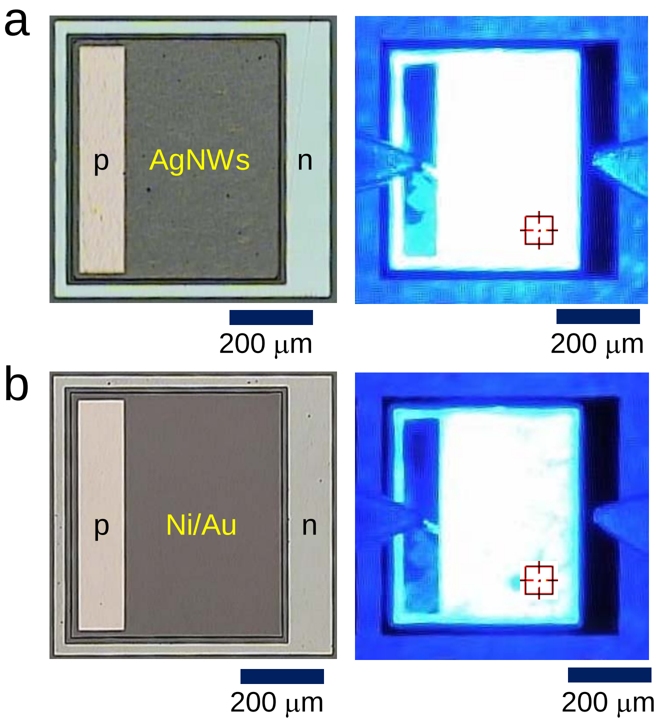

Supplementary Figure 4 EL images of AgNW-LEDs (a) and reference LEDs (b)

The EL images of standard LED structure with surrounding *n*-electrodes are shown (taken at 10 mA). The dimension of the mesa was 400 μm×500 μm. Despite the use of surrounding *n*-electrodes, the reference LEDs show still current crowding nearby *n*-electrodes, while the AgNWs LEDs show perfect current spreading.

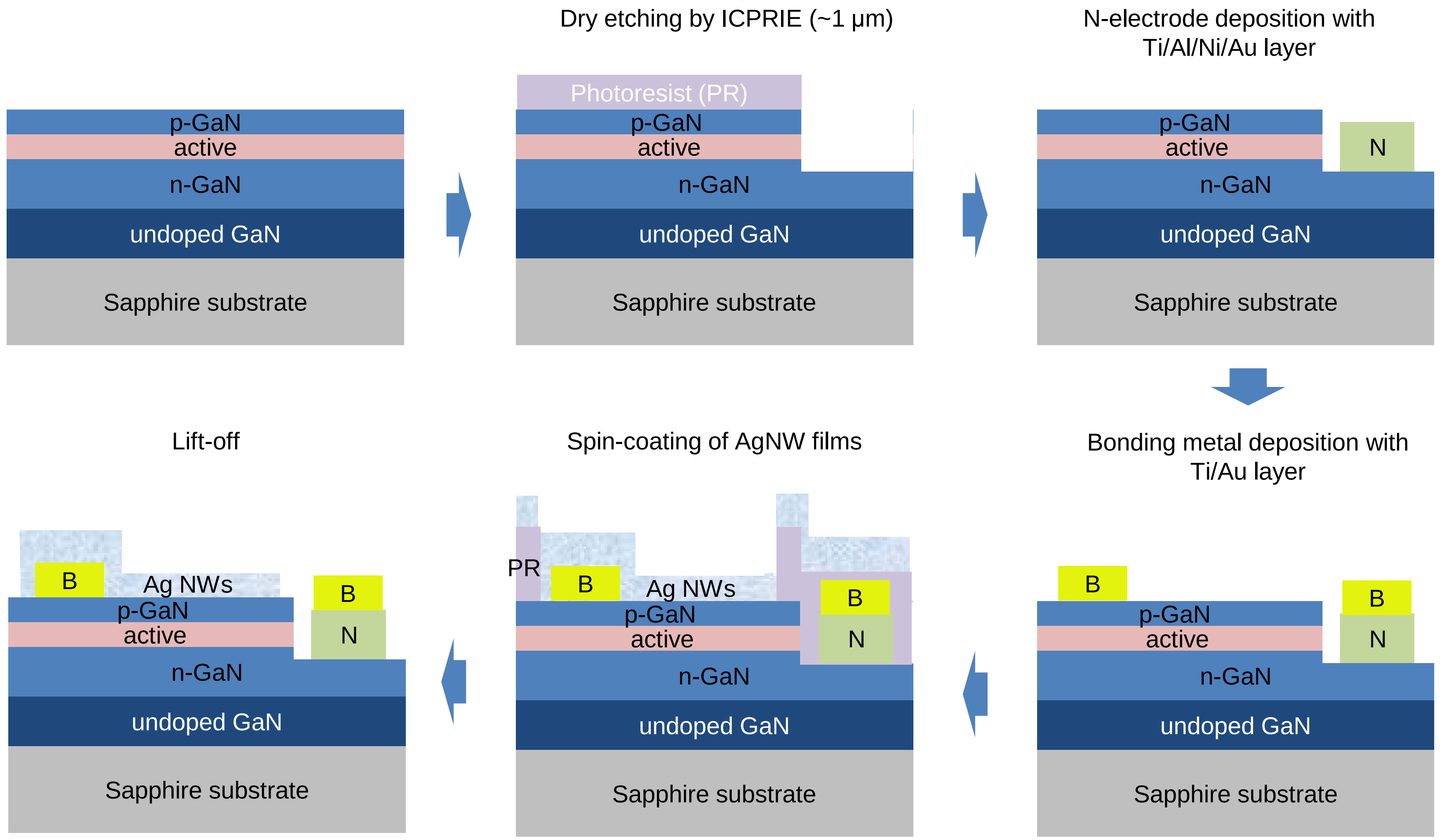

Supplementary Figure 5 Schematic fabrication procedure for LEDs with AgNWs TCEs

To fabricate LEDs with AgNW TCEs, the same method was used as that used to form the AgNW TLM pattern. For example, the rectangular mesa was defined by dry etching to a thickness of ~1.0 μm to expose the *n*-layer using an inductively coupled plasma reactive ion etching system, on which a Ti/Al/Ni/Au (30/70/30/70 nm) layer was deposited as an *n*-electrode by an *e*-beam evaporator. Rapid thermal annealing was performed at 550°C for 1 min in ambient N_2 to form an *n*-type ohmic contact. To form AgNW TCEs on the *p*-layer, first a Ti/Au (20 nm/10 nm) probing pad was formed on the mesa, followed by selective AgNW coating on the exposed *p*-layer and Ti/Au probing pads by means of a lift-off technique. It is noteworthy that the spin-coating process was performed in the last process step, which minimized possible contamination of AgNWs by additional photolithographic processing.
